# New insights into aflatoxin B1 mechanistic toxicology in cattle liver: an integrated approach using molecular docking and biological evaluation in CYP1A1 and CYP3A74 knockout BFH12 cell lines

**DOI:** 10.1007/s00204-024-03799-y

**Published:** 2024-06-04

**Authors:** Silvia Iori, Maija Lahtela-Kakkonen, Caterina D’Onofrio, Federica Maietti, Greta Mucignat, Anisa Bardhi, Andrea Barbarossa, Anna Zaghini, Marianna Pauletto, Mauro Dacasto, Mery Giantin

**Affiliations:** 1 Department of Comparative Biomedicine and Food Science, University of Padua, Viale Dell’Università 16, Legnaro, 35020 Padua, Italy; 2 School of Pharmacy, University of Eastern Finland, Yliopistonrinne 3, 70210 Kuopio, Finland; 3 Department of Veterinary Medical Sciences, Alma Mater Studiorum University of Bologna, Via Tolara di Sopra 50, Ozzano dell’Emilia, 40064 Bologna, Italy

**Keywords:** Aflatoxin B1, Bovine liver, CYP1A1, CYP3A74, Molecular docking, RNA-seq

## Abstract

Aflatoxin B1 (AFB1) is a pro-carcinogenic compound bioactivated in the liver by cytochromes P450 (CYPs). In mammals, CYP1A and CYP3A are responsible for AFB1 metabolism, with the formation of the genotoxic carcinogens AFB1-8,9-epoxide and AFM1, and the detoxified metabolite AFQ1. Due to climate change, AFB1 cereals contamination arose in Europe. Thus, cattle, as other farm animals fed with grains (pig, sheep and broiler), are more likely exposed to AFB1 via feed with consequent release of AFM1 in milk, posing a great concern to human health. However, knowledge about bovine CYPs involved in AFB1 metabolism is still scanty. Therefore, CYP1A1- and CYP3A74-mediated molecular mechanisms of AFB1 hepatotoxicity were here dissected. Molecular docking of AFB1 into CYP1A1 model suggested AFB1 8,9-*endo*- and 8,9-*exo*-epoxide, and AFM1 formation, while docking of AFB1 into CYP3A74 pointed to AFB1 8,9-*exo*-epoxide and AFQ1 synthesis. To biologically confirm these predictions, CYP1A1 and CYP3A74 knockout (KO) BFH12 cell lines were exposed to AFB1. LC–MS/MS investigations showed the abolished production of AFM1 in CYP1A1 KO cells and the strong increase of parent AFB1 in CYP3A74 KO cells; the latter result, coupled to a decreased cytotoxicity, suggested the major role of CYP3A74 in AFB1 8,9-*exo*-epoxide formation. Finally, RNA-sequencing analysis indirectly proved lower AFB1-induced cytotoxic effects in engineered cells *versus* naïve ones. Overall, this study broadens the knowledge on AFB1 metabolism and hepatotoxicity in cattle, and it provides the weight of evidence that CYP1A1 and CYP3A74 inhibition might be exploited to reduce AFM1 and AFBO synthesis, AFB1 toxicity, and AFM1 milk excretion.

**Supplementary Information:**

The online version contains supplementary material available at 10.1007/s00204-024-03799-y.

## Introduction

Aflatoxin B1 (AFB1) is a ubiquitous mycotoxin produced by *Aspergillus flavus* and *A. parasiticus*, contaminating a wide range of food and feed crops including cereals, oilseeds, tree nuts, and dried fruits. It is universally recognized that AFB1 poses a risk to human and animal health as a consequence of its hepatotoxic, genotoxic, and carcinogenic effects (Ostry et al. 2017; Mahato et al. 2019). At present, all the aflatoxins (AFs), including AFB1, aflatoxin B2 (AFB2), aflatoxin G1 (AFG1), aflatoxin G2 (AFG2), and the derivative aflatoxin M1 (AFM1) have been classified as group 1 carcinogens by the International Agency for Research on Cancer (IARC) (Ostry et al. 2017). Climate change is a potential emerging hazard, and it is a common opinion it can increase the risk of AFs contamination. Indeed, variations in climate-related abiotic factors such as higher temperature, water stress conditions (e.g., changes in water activity), and increasing levels of atmospheric carbon dioxide (CO_2_) can enhance *Aspergillus spp.* growth and AFs biosynthesis also in European temperate areas, leading to increased levels of contamination in agricultural commodities and derived food and feed products (Duchenne-Moutien and Neetoo 2021). Aflatoxin B1 is still considered the most toxic AF and one of the most potent liver genotoxic carcinogen. As to the other AFs, AFG1 is slightly less genotoxic than AFB1, while AFB2 and AFG2 are much less genotoxic than AFB1 (EFSA 2020). AFG1 and AFM1 are even carcinogenic, although AFM1 is a tenfold less potent hepatic carcinogen than AFB1. For AFB2 and AFG2, there is limited or inadequate evidence about their carcinogenicity (EFSA 2020).

The cytochrome P450 (CYP) superfamily of xenobiotic metabolizing enzymes (XMEs) are the hinge of hepatic AFB1 biotransformation/bioactivation and produce a number of derivatives depending on the biotransformation pathway. In human and other mammalian or avian susceptible species, CYP1A and CYP3A subfamilies catalyze the 8,9-epoxidation of AFB1, giving rise to AFB1 8,9-*endo*- and 8,9-*exo*-epoxide (AFBO) derivatives. The latter metabolite forms adducts with DNA (AFB1-N7-guanine and AFB1-formamidopyrimidine) causing potential G-to-T transversions, and with lysine residues on serum albumin via AFB1 dihydrodiol (AFBO hydrolytic product) and its base-catalyzed rearrangement to a dialdehyde (EFSA 2007), leading to toxicity, impairment of transcriptional processes, and initiation of carcinogenesis (Bedard and Massey 2006). Furthermore, CYP1A- and CYP3A-mediated hydroxylation reactions result in the formation of the genotoxic carcinogenic AFM1 and the relatively nontoxic aflatoxin Q1 (AFQ1), respectively (Wang et al. 2022). Additional metabolites are the genotoxic/carcinogenic aflatoxicol (AFL), synthesized by a NADPH-dependent reductase (Murcia and Diaz 2020), and the relatively nontoxic derivatives aflatoxin P1 (AFP1) and AFB2 produced by CYPs (Donhal et al. 2014). Noteworthy, AFL, whose carcinogenic potency was proven to be lower than AFB1 (EFSA 2020), may be converted back to AFB1 by a microsomal dehydrogenase, thus increasing AFB1 permanence in the body (Karabulut et al. 2014; Wang et al. 2022). In human, the hepatic CYP3A4 and CYP1A2 are the first and foremost XMEs responsible for AFB1 metabolism, preferentially leading to different types of derivatives; AFBO and AFQ1 are produced by CYP3A4, while CYP1A2 gives rise to both epoxides and AFM1 (Gallagher et al. 1996; Wang et al. 1998). Species differences in the constitutive expression and activity of CYP isoforms as well as of hepatic glutathione S-transferases (GSTs), a family of conjugative XMEs primarily involved in the detoxification of AFB1 and its derivatives (Dohnal et al. 2014; Deng et al. 2018), are responsible for a different susceptibility to AFB1. Indeed, the extreme sensitivity of turkeys to AFB1 has been associated to the high activity of CYP1A5 and CYP3A37 and the deficient GST-mediated conjugative pathway (Rawal and Coulombe 2011). The trout is known as the most sensitive species to AFs (Santacroce et al. 2008). Ruminants are relatively more resistant to AFs than monogastric animals (i.e., pigs) as the rumen microflora acts as a first line of defense against mycotoxins (Fink-Gremmels 2008a; Popescu et al. 2022). Nevertheless, AFs are only partly degraded by the ruminal flora, with AFL as a typical secondary metabolite of rumen metabolism (Fink-Gremmels 2008a), and interfere with the overall ruminal digestive capacity (Gallo et al. 2015).

Cattle are an important food-producing species, and cow milk one of the most valuable foods for humans. When dairy cows are fed with AFB1-contaminated feed, even at concentrations approaching European regulatory levels, measurable amounts of AFM1 as well as traces of AFL are found in dairy milk (EFSA 2004; Fink-Gremmels 2008a), consequently posing a risk to human health (Min et al. 2021). Beyond all this, cattle exposure to high AF concentrations or mixtures impairs liver function, compromises immune function, and reduces feed intake, which altogether might explain the impact on growth performances, milk production, and susceptibility to diseases (Fink-Gremmels 2008b; Jiang et al. 2021). Moreover, AFB1 can affect reproductive function by reducing viability and DNA integrity of bull sperm and causing damage to the bovine preimplantation embryo (reviewed in Jiang et al. 2021). As a consequence, AFs represent also an economic issue for dairy cattle industry.

The mycotoxin-induced hepatotoxicity has been recently characterized in a bovine fetal hepatocyte cell line (BFH12) using RNA-sequencing (RNA-seq) and confirmatory analyses at protein level; overall, a dysregulation of genes mainly involved in inflammatory and oxidative stress responses, xenobiotic metabolism, apoptosis, and cancer was highlighted (Iori et al. 2022). In addition, the presence of a molecular pathway linking oxidative stress and inflammation has been postulated, with a pivotal role played by the toll-like receptor 2 (TLR2), that resulted in upregulation following AFB1 exposure (Iori et al. 2022). Compared to human species, knowledge about the CYP isoenzymes taking part in the biotransformation of AFB1 in cattle is still limited. A previous in vitro study highlighted the role of CYP3A and CYP1A subfamilies in AFB1 metabolism in bovine primary hepatocytes (Kuilman et al. 2000). Moreover, the gene expression of CYP3A74 (human CYP3A4-like) and CYP1A1 has been shown to be upregulated by AFB1 in a bovine hepatocyte-like cell line, suggesting a central role of these isoenzymes in AFB1 biotransformation as well as a putative role of AFB1 as CYP1A and CYP3A inducer (Iori et al. 2022). To address such a lack of knowledge, in the present study, we performed molecular docking investigations to examine the potential interactions of AFB1 with bovine CYP1A1 and CYP3A74 apoproteins. To confirm the obtained in silico data, in vitro studies were, therefore, carried out on *CYP1A1* knockout (KO) and *CYP3A74* KO hepatocyte-like cell lines (BFH12) recently obtained and characterized in our lab (Iori et al. 2024a, b). Briefly, engineered cells were exposed to AFB1 sub-cytotoxic concentrations, and the specific contribution of both CYPs to the mycotoxin biotransformation was assessed using a liquid chromatography coupled to tandem mass spectrometry (LC–MS/MS) protocol. To better characterize the AFB1 biological effects on engineered cells, we measured lipid peroxidation, AFB1 cytotoxicity, and assessed the resulting transcriptomic changes by RNA-seq. Overall, this integrated approach allowed us to gain new insights into CYP1A1 and CYP3A74 involvement in the molecular events underpinning AFB1 hepatotoxicity in cattle.

## Materials and methods

### Chemicals and reagents

Dexamethasone, insulin from bovine pancreas and AFB1 were from Sigma-Aldrich (St. Louis, MO, USA). The WST-1 Cell Proliferation Reagent was from Roche (Basel, Switzerland). Complete William’s E Medium, fetal bovine serum (FBS), penicillin/streptomycin solution, Qubit RNA Assay Kit and BCA Assay Kit were from Thermo Fisher Scientific (Waltham, Massachusetts, USA). RNeasy Mini kit was from Qiagen (Hilden, Germany). Analytical standards of AFB1, AFL, AFM1, AFQ1, and aflatoxin M2 (AFM2, used as internal standard) were purchased from TRC Canada (North York, ON, Canada). The rabbit anti-human TLR2 (A2545), AKT3 (A12909) and P21 (A1483) primary antibodies were from ABclonal (Woburn, MA, USA). The mouse anti-human MAPK11 (F-3; sc-390984) was obtained from Santa Cruz Biotechnology (Dallas, TX, USA). The rabbit anti-beta actin (ACTB, GTX109639), the goat anti-rabbit IgG, and the goat anti-mouse IgG were from GeneTex (Irvine, CA, USA).

### Homology modeling and molecular docking of AFB1 into CYP1A1 and CYP3A74 models

Bovine CYP1A1 and CYP3A74 models were obtained from human CYP1A1 and CYP3A4 crystal structures published in Walsh et al. 2013, and Sevrioukova and Poulos 2017. Molecular modeling was performed using the Schrödinger Maestro version 12.8 (Small-Molecule Drug Discovery Suite 2021–2, Schrödinger, LLC, New York, NY). Co-crystallized ligands (i.e., alpha-naphthoflavone and midazolam for CYP1A1 and CYP3A4, respectively) as well as AFB1 were docked into the CYPs using Glide SP. Additional details are reported in the Supplementary file.

### Cell culture

Cell models used in this experiment, namely CTL (control), CYP1A1 KO, and CYP3A74 KO, were established and characterized in previous works (Iori et al. 2024a, b). The conditions of cell line maintenance have been reported elsewhere (Pauletto et al. 2020).

#### Cells incubation

For cytotoxicity screening, cells were seeded in 96-well culture plates at a density of 6 × 10^3^ cells/well, and 4 days post-plating, they were exposed for 48 h to three AFB1 concentrations (i.e., 0.9, 1.8, and 3.6 μM) dissolved in medium containing 0.1% dimethylsulfoxide (DMSO). Cells exposed to the vehicle alone were used as control. Aflatoxin B1 sub-cytotoxic concentrations were defined according to a precedent dose–response curve (Pauletto et al. 2020), and corresponded to those used in a previous published study (Iori et al. 2022). Cell viability was determined using the WST-1 Cell Proliferation Reagent, following the manufacturer’s instructions. Three biological replicates per experimental group were performed, and each concentration was tested in sextuplicate.

Cells were seeded in six-well culture plates (density 5 × 10^4^ cells/well) and 90-mm Petri dishes (density 3 × 10^5^ cells/Petri dish) for gene expression and LC–MS/MS investigations, and confirmatory assays, respectively. Cells were then exposed either to AFB1 (0.9 and 1.8 μM) or DMSO following the same experimental protocol described above. After 48 h of incubation, the medium was collected and stored at -80 °C for analytical investigations, while cell monolayers were collected as previously described (Iori et al. 2022). Three independent experiments were executed.

### LC–MS/MS quantification of AFB1, AFM1, AFL, and AFQ1

AFB1 and its metabolites (AFM1, AFL, and AFQ1) were quantified by LC–MS/MS in the medium of CTL and KO cells treated with 0.9 and 1.8 µM AFB1. Methodological details are provided in the Supplementary File. The amount of AFB1 and its derivatives was calculated by dividing the amount of the metabolite in the culture medium by the total protein content of the respective cell monolayer. Three biological replicates per experimental group were performed.

### Total RNA extraction and RNA-sequencing analysis

Total RNA was isolated from cells as previously described (Iori et al. 2022). Library construction (QuantSeq 3' mRNA-Seq Library Prep Kit FWD, Lexogen GmbH, Austria) and sequencing (Illumina Novaseq 6000, single-end 75 bp) were performed at the NGS facility of the Department of Biology (University of Padua, Italy). Three biological replicates per experimental group were considered. Raw reads trimming and mapping, as well as differential gene expression analysis were conducted as reported in the Supplementary File. The differentially expressed genes (DEGs) resulting from *CYP1A1* and *CYP3A74* gene KO (described in Iori et al. 2024a and Iori et al. 2024b) were filtered out, and a functional analysis (detailed in the Supplementary file) was finally conducted on DEGs modulated exclusively by AFB1.

### Total proteins isolation and immunoblotting

Total proteins were extracted from cell monolayers as detailed in Iori et al. (2022), and quantified using the BCA assay kit. Protein samples from CTL and KO cells exposed to 1.8 µM AFB1 were then subjected to immunoblotting investigations for P21, TLR2, MAPK11, and AKT3 proteins (Supplementary file).

#### Oxidative stress (lipid peroxidation)

To measure oxidative stress, lipid peroxidation (malondialdehyde production) was evaluated in CTL and KO cells exposed to 1.8 µM AFB1. The amount of malondialdehyde (MDA) was quantified in undiluted cell lysates (total proteins) using the ab233471 lipid peroxidation (MDA) colorimetric assay kit (Abcam, Prodotti Gianni S.p.A., Milan, Italy). Each sample was assayed in duplicate, following manufacturer’s instructions and using a Multiskan GO multiwell plate reader (Thermo Fisher Scientific, Waltham, MA, USA).

### Statistical analysis

The statistical analysis was performed using GraphPad Prism software (version 9.5.1, San Diego, CA, USA). One-way ANOVA followed by Dunnett’s multiple comparisons test was implemented. A value of *p* < 0.05 was considered as statistically significant.

## Results

### Homology modeling and molecular docking of AFB1 into CYP1A1 and CYP3A74 models

The sequence identity between human CYP1A1 and bovine CYP1A1 was 81%; the one between human CYP3A4 and bovine CYP3A74 was 77% (Supplementary Fig. 1a, b). The orientation of co-crystalized ligands in the binding pocket of cattle CYP1A1 and CYP3A74 models was identical to that into templates, suggesting that the docking parameters were suitable for the final docking studies of AFB1 against bovine CYPs of interest (data not shown). Worth noting, only docked poses for which the site of metabolism (SOM) was within 6 Å from the heme iron were considered (Keizers et al. 2005).

**Fig. 1:**
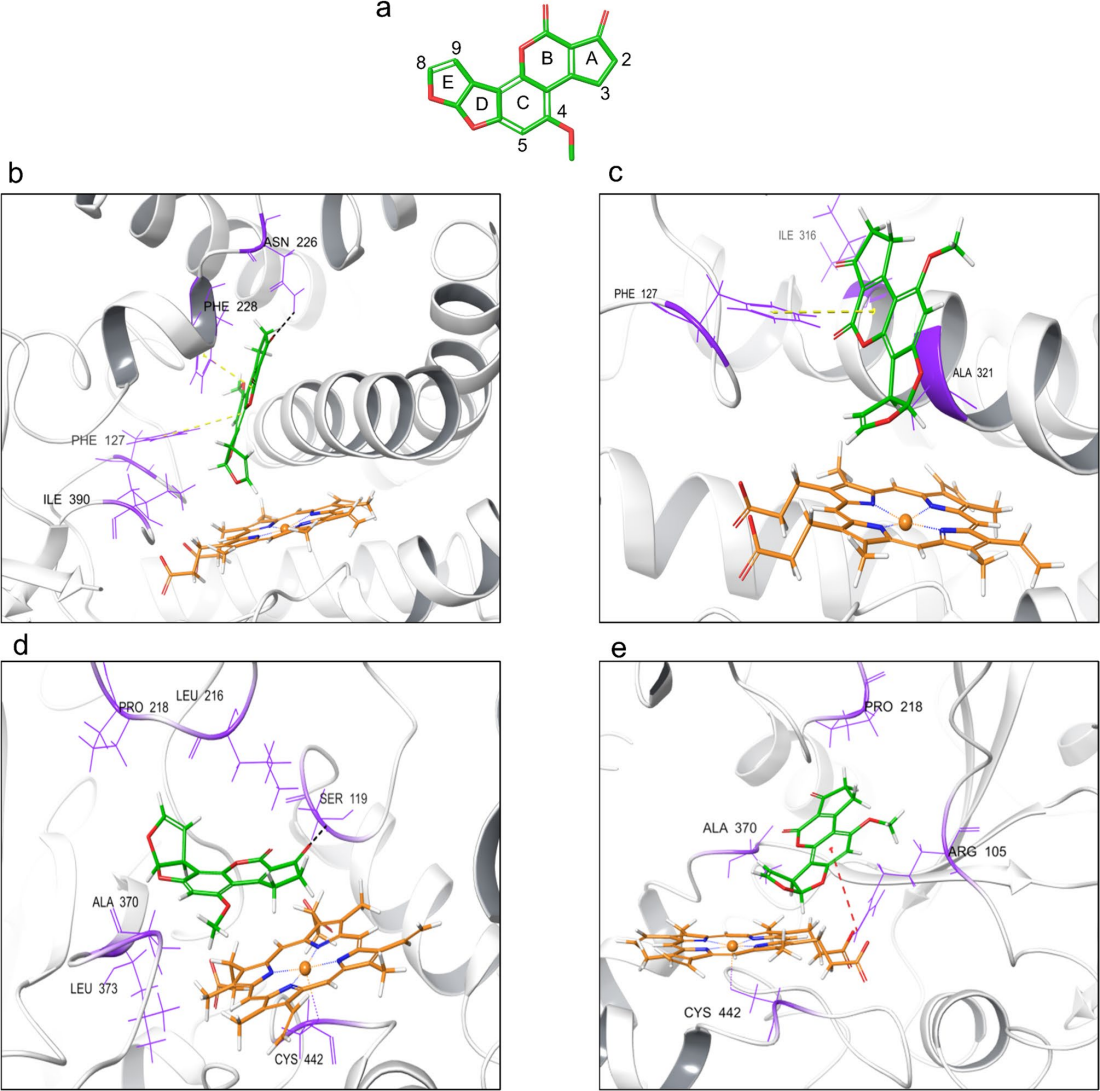
AFB1 structure (**a**) and docking poses of AFB1 against CYP1A1 (**b**, **c**) and CYP3A74 (**d**, **e**) models. CYPs backbone is shown in grey ribbon, while hydrophobic residues that surround the ligand are figured in violet. Black dashes indicated hydrogen bonds, while yellow and red dashes indicated π–π stacking and π–cation interactions, respectively

As to CYP1A1, two possible AFB1 binding modes were highlighted (Fig. 1b, c). A first docking pose (Fig. 1b; docking score-DC − 8.4) disclosed a hydrogen bond between ASN 226 and the carbonyl oxygen on ring A, with an interatomic distance between C8 and Fe^3+^ of 3.94 Å. Looking deeper, AFB1 was located in the binding pocket near hydrophobic residues such as ILE 390. What is more, π–π stacking interactions between PHE 228 and PHE 127 and B and C rings were revealed, respectively. A further docking orientation (Fig. 1c; DC − 8.0) showed that AFB1 was placed near hydrophobic residues corresponding to ILE 316 and ALA 321. A closer investigation revealed π–π stacking interactions between PHE 127 and B ring, with a distance between C8 and Fe^3+^ equal to 4.75 Å.

Two docking poses of AFB1 against CYP3A74 model were highlighted (Fig. 1d, e). In the first one (Fig. 1d; DC − 6.4), a hydrogen bond between the SER 119 and the carbonyl oxygen on ring A was unveiled. Specifically, the interatomic distance between C3 and heme iron was 3.92 Å. In addition, several hydrophobic residues such as PRO 218, ALA 370, LEU 373, and LEU 216 surrounded AFB1, thus potentially favoring the establishment of others hydrogen bonds. The second docking pose (Fig. 1e; DC − 6.3) revealed a π–cation interaction between ARG 105 and the ring C, with an interatomic distance between C8 and heme iron of 4.80 Å. Moreover, hydrophobic residues like PRO 218 and ALA 370 encircling AFB1 were predicted.

### LC–MS/MS quantification of AFB1, AFM1, AFL, and AFQ1

**Fig. 2:**
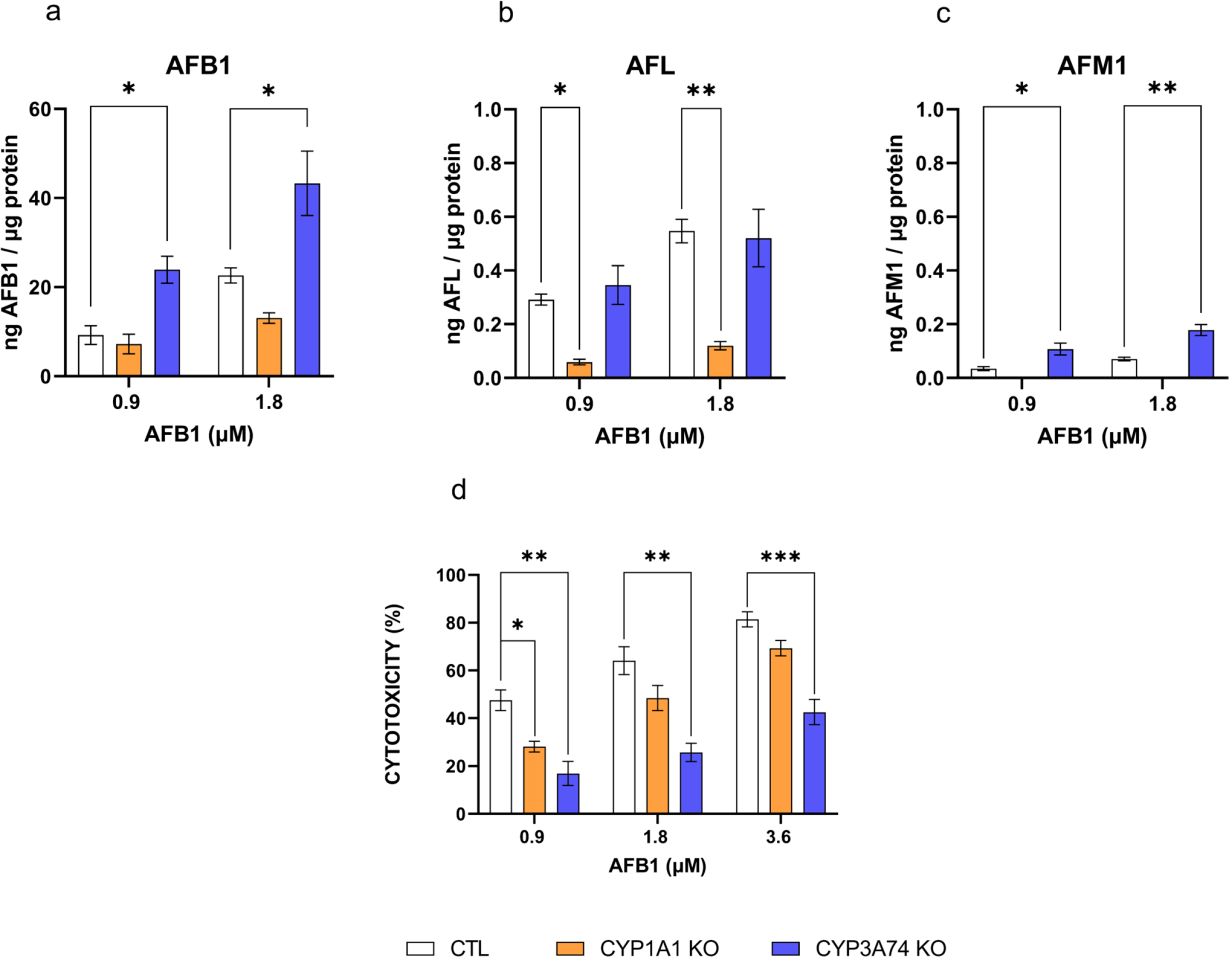
Amount of AFB1 (**a**), AFL (**b**), and AFM1 (**c**) detected by LC–MS/MS in the medium of CTL, CYP1A1 KO, and CYP3A74 KO cells exposed to 0.9 and 1.8 µM AFB1 and normalized to the total protein content of the respective cell monolayers. Data are expressed as ng of analyte per µg of total protein, as the mean ± mean standard error (SEM) of three biological replicates. Cytotoxicity evaluation (**d**) of increasing AFB1 concentrations in CTL, CYP1A1 KO, and CYP3A74 KO cells using WST-1 reagent. Data are expressed as the mean percentage of dead cells relative to that of cells exposed to the vehicle only (0.1% DMSO) ± SEM of three biological replicates, each performed in sextuplicate. Statistical analysis: one-way ANOVA followed by Dunnett’s multiple comparisons test; *: *p* < 0.05, **: *p* < 0.01, and ***: *p* < 0.001

Control cells exposed to AFB1 produced detectable amounts of AFL and AFM1 derivatives in accordance with the concentration used (Fig. 2a–c), confirming the capability of BFH12 cells to metabolize AFB1 in a dose-dependent manner (Pauletto et al. 2020; Iori et al. 2022). Conversely, AFQ1 metabolite was never detected in both CTL and genetically modified cells (data not shown). As expected, in CYP1A1 KO and CYP3A74 KO cells, the AFB1 metabolite profiling appeared modified. In detail, in CYP1A1 KO cells, the amount of AFB1 detected in the cell medium was comparable to the one quantified in CTL cells, suggesting a still active AFB1 metabolism in the CYP1A1 KO cell model. However, AFL amounts dropped to ~ 20% (*p* < 0.05), and AFM1 was below the limit of detection for both AFB1 concentrations (Fig. 2b, c). Conversely, in CYP3A74 KO cells, the concentration of the parent compound in the medium was twice the one of CTL (*p* < 0.05, Fig. 2a) for both AFB1 concentrations, thus suggesting a reduced biotransformation of the mycotoxin in cells lacking CYP3A74. In this respect, the amount of AFL did not change in the engineered cells compared to CTL ones, while that of AFM1 was significantly increased (~ threefold) in a dose-dependent manner (*p* < 0.05, Fig. 2b, c).

### Aflatoxin B1 cytotoxicity

Overall, both CTL and genetic modified cells showed a dose-dependent increase of cell death due to AFB1 (Fig. 2d); nevertheless, engineered cells were less sensitive to AFB1 compared to CTL, albeit with some differences. Specifically, in CYP1A1 KO cells, the cytotoxicity of AFB1 was only slightly reduced by gene ablation compared to CTL cells (~ 20% (*p* < 0.05), ~ 15% and ~ 12% at 0.9, 1.8 and 3.6 µM AFB1, respectively). The lack of *CYP3A74* gene caused a lower cytotoxicity at all the tested concentrations (~ 30%, ~ 40%, and ~ 40% at 0.9, 1.8 and 3.6 µM AFB1, respectively; *p* < 0.01).

### Transcriptome analysis

A total of 56,172,201 and 57,631,257 raw reads were obtained when comparing CYP1A1 KO and CYP3A74 KO cells with CTL ones, respectively. Raw reads were deposited in GeneBank under the BioProject accession number ID PRJNA1068490. The plot MDS provided an unsupervised clustering of samples (data not shown). Concerning CYP1A1 KO, a total of 1521 and 1505 DEGs were highlighted at 0.9 and 1.8 μM AFB1, respectively. As to CYP3A74 KO cells, 190 and 253 DEGs were identified at 0.9 μM and 1.8 μM AFB1, respectively. The whole list of DEGs resulting from each comparison is reported in the Supplementary Table 1a, b. For each comparison, a Venn diagram was built up to visualize unique and shared DEGs after AFB1 treatment (Supplementary Fig. 2a, b). As a confirmation of the dose-dependent effects of AFB1, about 81% and 91% of DEGs modulated by the lowest concentration overlapped with the DEGs affected by the highest concentration in CYP1A1 KO and CYP3A74 KO cells, respectively. Based on this result, the subsequent functional analysis was performed uniquely on the DEGs modulated by 1.8 μM AFB1 (i.e., 1505 and 253 for CYP1A1 KO and CYP3A74 KO, respectively), assuming that the transcriptome perturbations induced by the highest concentration better characterize the overall effects of AFB1 in the two KO cell models.

### Functional analysis of DEGs

The functional analysis of the DEGs modulated by 1.8 μM AFB1 in CYP1A1 KO cells *vs* CTL revealed the enrichment of four pathways (Supplementary Fig. 3a) which were mainly related to cell proliferation (i.e., ‘PD-L1 expression and PD-1 checkpoint pathway in cancer’, ‘AGE-RAGE signaling pathway in diabetic complications’, ‘Choline metabolism in cancer’ and ‘Relaxin signaling pathway’). The list of genes whose expression was induced in CYP1A1 KO cells exposed to AFB1 included the SOS Ras/Rho guanine nucleotide exchange factor 1 (*SOS1*) and 2 (*SOS2*), the KRAS proto-oncogene, GTPase (*KRAS*), the AKT serine/threonine kinase 3 (*AKT3*) as well as the mechanistic target of rapamycin kinase (*MTOR*). Furthermore, the G protein subunit beta 4 (*GNB4*) and gamma 12 (*GNG12*), the collagen type IV alpha 2 (*COL4A2*) and 3 (*COL4A3*) chain, as well as the cyclin D1 (*CCND1*) were upregulated in CYP1A1 KO cells. Among genes resulting downregulated in AFB1-exposed CYP1A1 KO cells, we highlighted the toll-like receptor 2 (*TLR2*) and the mitogen-activated protein kinase 11 (*MAPK11*), together with the vascular endothelial growth factor B (*VEGFB*) and two genes encoding for diacylglycerol kinase proteins (i.e., *DGKA* and *DGKD*).

Focusing on CYP3A74 KO cells, the output of the analysis highlighted ten enriched pathways (Supplementary Fig. 3b). Several pathways encompassed genes linked to inflammatory processes (e.g., ‘Cytokine-cytokine receptor interaction’, ‘Rheumatoid arthritis’, ‘Chemokine signalling pathway’, ‘NF-kappa B signalling pathway’). These pathways included genes whose mRNA levels resulted inhibited in engineered cells, such as the chemokine (C–C motif and C-X-C motif) ligand 2 (*CCL2* and *CXCL2,* respectively), the matrix metallopeptidase 1 (*MMP1*), the intercellular adhesion molecule 1 (*ICAM1*), and the prostaglandin-endoperoxide synthase 2 (*PTGS2*). Some KEGG pathways related to cell cycle regulation and growth (e.g., ‘Lipid and atherosclerosis’, ‘Vascular smooth muscle contraction’, ‘TNF signalling pathway’) were over represented, too. Among the upregulated genes, we noticed the very low-density lipoprotein receptor (*VLDLR*) as well as the potassium calcium-activated channel subfamily M alpha 1 (*KCNMA1*). However, several genes were downregulated in CYP3A74 KO cells exposed to AFB1, such as the melanoma growth stimulating activity alpha (*GRO1*), the cyclin-dependent kinase inhibitor 1A (*CDKN1A/P21*), the calcium voltage-gated channel subunit alpha1 F (*CACNA1F*) and A-Raf proto-oncogene, serine/threonine kinase (*ARAF*).

### Confirmatory assays: lipid peroxidation and immunoblotting

When exposed to 1.8 µM AFB1, CYP1A1 KO cells showed a significant increase in MDA production compared to CTL cells (~ fourfold; *p* < 0.001); conversely, MDA amount was comparable in CYP3A74 KO and CTL cells (Supplementary Fig. 4). Immunoblotting investigations (Supplementary Fig. 5) confirmed mRNA expression data, even if statistically significant differences were obtained for MAPK11 only (*p* < 0.01, CYP1A1 KO *vs* CTL cells).

## Discussion

Humans and all animal species are susceptible to aflatoxicosis. Human and rat are more sensitive to AFB1 than mouse, which is very tolerant to this mycotoxin (Donhal et al. 2014). In poultry species, the order of sensitivity to AFB1 is duck > turkey > Japanese quail > chicken (Monson et al. 2015). As to aquatic species, the trout is considered the most sensitive species to AFB1 (Santacroce et al. 2008). Among ruminants, that generally are more resistant to aflatoxicosis than monogastric animals (i.e., pigs) thanks to the rumen microflora (Fink-Gremmels 2008a; Popescu et al. 2022), cattle are more sensitive than sheep; additionally, beef and dairy cattle are more susceptible to AFB1 than horses (Radostits et al. 2007). Differences in response to AFs include the proportion of AF metabolized by CYPs into AFBO compared to other much less toxic metabolites, and the prevalence of pathways that lead to the formation of nontoxic conjugates with lower mutagenicity and cytotoxicity (Donhal et al. 2014). Thus, from a general point of view, the study of the key enzymes involved in AFB1 metabolism is fundamental to get more insights into the species-specific toxicity and response to the mycotoxin.

Up to present, the knowledge about the role of bovine CYPs in AFB1 metabolism is still limited. The only available information date back to 2000, when Kuilman and colleagues suggested the contribution of both CYP1A and CYP3A subfamilies in the production of AFM1 and AFB1-dihydrodiol in bovine primary hepatocytes (Kuilman et al. 2000). More recently, we observed a transcriptional modulation of *CYP1A1* and *CYP3A74* genes in BFH12-naïve cells exposed to AFB1 increasing concentrations; therefore, we postulated that these isoenzymes play an important role in AFB1 hepatic metabolism in cattle (Pauletto et al. 2020; Iori et al. 2022). In the present study, we used CYP1A1 KO and CYP3A74 KO cells (Iori et al. 2024a, b) and an integrated approach encompassing targeted molecular docking, AFB1 metabolite profiling, cytotoxicity, and the assessment of the related transcriptional changes, to ascertain the role of these two important CYP isoforms in the molecular events underpinning AFB1 hepatotoxicity in cattle.

Molecular docking results clearly showed that both CYP1A1 and CYP3A74 models may accommodate AFB1 in their binding pocket. As to CYP1A1 apo-structure, two putative binding poses were predicted. In the first one, the 8,9-*endo*-epoxide metabolite formation seemed to be the favorite one, as the AFB1 *endo* side of the C8–C9 double bonds was oriented toward the porphyrin ring. In a second binding pose, the E ring of AFB1 faced the heme iron, thus assuming an orientation that might predispose to the formation of both the AFB1 9α-hydroxy metabolite (i.e., AFM1), and the AFB1 8,9-*exo*-epoxide. Concerning CYP3A74 model, two potential catalytically active conformations were highlighted. In the first one, the C3 was close to the heme iron catalytic center, potentially leading to the formation of the less toxic AFQ1 (AFB1 3α-hydroxy metabolite). In the second pose, the *exo* side of the C8–C9 double bonds faced the catalytic center, thereby assuming an orientation prone to the formation of the AFB1 8,9-*exo*-epoxide. Overall, cattle molecular docking studies predictions confirmed those previously described for humans (Bonomo et al. 2017), thereby suggesting the involvement of both bovine CYPs in hepatic AFB1 metabolism.

To confirm the above mentioned in silico predictions, the AFB1 metabolite profiling was investigated in CTL, CYP1A1 KO and CYP3A74 KO cells using a LC–MS/MS protocol. As previously reported (Pauletto et al. 2020; Iori et al. 2022), CTL cells metabolized AFB1 in a dose-dependent manner, and released detectable amounts of AFL and AFM1 into the medium. However, in CYP1A1 KO cells, the production of AFM1 was completely abolished, confirming the main role of bovine CYP1A1 in AFB1 9α-hydroxylation, as predicted by molecular docking in cattle and humans, too (Bonomo et al. 2017). Interestingly, also AFL synthesis was strongly reduced in cells deprived of CYP1A1 apoprotein. This result might be indirectly associated to *CYP1A1* deletion; indeed, *AKR1A1,* an aldo–keto reductase putatively responsible of AFB1 enzymatic reduction at the ketonic carbonyl group (Karabulut et al. 2014), was downregulated in BFH12 cells as a consequence of the genetic modification (Iori et al. 2024a). Despite the drop of AFM1 and AFL, AFB1 was still metabolized in CYP1A1 KO cells, and its concentration was comparable to the one detected in CTL cells. In our opinion, this finding might be due to the persistent constitutive expression of CYP3A74, along with CYP1B1, whose involvement in AFB1 metabolism has been previously reported in humans (Chen et al. 2023) and confirmed in BFH12 cells, too (Iori, personal data). Based on cytotoxicity results, showing only a slight decrease of cell mortality in CYP1A1 KO *vs* CTL cells, we infer that CYP3A74 actively participates in AFB1 metabolism both in AFB1 epoxidation (as predicted by molecular docking) and in the production of further toxic derivatives.

Other clues, supporting CYP3A74 hinge role in AFB1 metabolism, arise from CYP3A74 KO cells results. Indeed, the significant accumulation of the unmetabolized AFB1 coupled to the lowest cytotoxicity observed in the cells deprived of *CYP3A74* gene would suggest a major contribution of CYP3A74 in AFB1 bioactivation (i.e., AFBO synthesis) rather than in its detoxification (AFQ1). This assumption is further strengthened by the evidence of BFH12 cells inability to produce AFQ1, since this metabolite was never detected in both CTL and CYP3A74 KO cells. In addition, the countertrend increase in AFM1 production might be ascribed to CYP1A1; indeed, the latter was significantly induced in CYP3A74 KO cells, probably because of a compensatory effect (Iori et al. 2024b).

To deepen CYP1A1- and CYP3A74-mediated molecular mechanisms underpinning AFB1 hepatotoxicity, transcriptomic perturbations resulting from AFB1 exposure in engineered cells were evaluated by RNA-seq. Our previous study on BFH12-naïve cells pointed out the impact of AFB1 on several biological pathways such as inflammation, oxidative stress, drug metabolism, apoptosis, and cancer (Iori et al. 2022). However, in the present study, only few pathways were modulated by AFB1, suggesting that the deletion of both CYPs has a counteracting effect on AFB1 toxicity.

Going more into details, in CYP1A1 KO cells, several members of the KRAS/AKT/mTOR pathway, a signaling transduction network which promotes cell survival, cell cycle progression, and cell growth (Zhang et al. 2020), were dysregulated by AFB1. Specifically, two genes (*SOS1* and *SOS2*) responsible of *KRAS* activation and the initiation of a downstream signaling cascades involving AKT-mTOR axis (Sheffels et al. 2018; Zhang et al. 2020) were induced upon AFB1 treatment, together with *AKT3* (whose increased expression was confirmed also at the protein level) and *MTOR*, possibly promoting cell proliferation and survival. In addition, increasing mRNA levels of genes encoding for collagenase proteins (i.e., *COL4A2* and *COL4A3*) were noticed; these ones are two of the major structural components of the tumor microenvironment in hepatocellular carcinoma (HCC) together with *CCND1*, a gene overexpressed in multiple malignant tumors, including HCC itself (Liu et al. 2020; Ding et al. 2020). On the other hand, the *VEGFB* gene was downregulated; this vascular endothelial growth factor controls tissue lipid accumulation, and its inhibition reduces hepatic steatosis and non-alcoholic fatty liver disease in mice (Falkevall et al. 2023). Two further downregulated genes were *DGKA* and *DGKD,* which are involved in the conversion of diacylglycerol to phosphatidic acid. This result is suggestive of a disbalance in lipid homeostasis (Massart and Zierath 2019) in CYP1A1 KO cells exposed to AFB1. Worth noting, two pivotal upregulated genes previously hypothesized to be involved in a pathway linking TLR2 activation to AFB1-hepatotoxicity, namely *TLR2* and *MAPK11* (Iori et al. 2022), showed here an opposite behavior (i.e., downregulation), confirmed also at the protein level. Accordingly, we might infer that *CYP1A1* deletion might have somehow reduced AFB1-mediated TLR2 activation and consequently the inflammatory response triggered by MAPK11, as also suggested by CYP1A1 KO cells cytotoxicity results.

Concerning CYP3A74 KO cells, the functional analysis highlighted the dysregulation of genes associated with inflammatory processes and cell cycle regulation. Noteworthy and compelling, all the DEGs pointed out by the functional analysis overlapped with those modulated by AFB1 in BFH12-naïve cells (Iori et al. 2022), but the trend of expression was completely opposite. Among these ones, a gene responsible of the prostanoid biosynthesis (*PTGS2*) as well as two chemokines (*CCL2* and *CXCL2*) were downregulated, thus indicating a decreased AFB1-mediated stimulation of inflammation in CYP3A74 KO cells. Likewise, decreased mRNA levels compared to CTL were noticed for *CDKN1A* (the gene encoding for the P21 protein). This protein, whose decreased trend of expression was confirmed by immunoblotting investigations, plays an outstanding role in cell cycle regulation, particularly in cell cycle arrest upon genotoxic damage (Ticli et al. 2022); therefore, this result would suggest that *CYP3A74* deletion might have a “protective role”, reducing the DNA damage caused by AFB1; again, this result indirectly confirms the fundamental role of CYP3A74 in AFB1 epoxidation. Interestingly, also the oncogene *GRO1*, commonly upregulated in a number of tumors including HCC (Huang et al. 2021) was downregulated in CYP3A74 KO cells. This evidence would confirm previous data showing that the inhibition of *GRO1* may lead to a decline of liver cancer cells proliferation through the cell cycle arrest (Huang et al. 2021). Another downregulated DEG was *MMP1*, the gene encoding for collagenase-1, whose upregulation has been previously associated with liver tissue damage in mice fed with an AFB1-contaminated diet (Al-Mudallal 2023). Finally, two further downregulated genes were *ICAM1*, coding for CD54 protein driving tumor growth (Benedicto et al. 2017) and *ARAF*, whose overexpression in HCC initiation and progression has been previously demonstrated (Ranjpour et al. 2019). Among genes upregulated in CYP3A74 KO cells, worth of mention are *VLDLR* and *KCNMA1*; the upregulation of the former was previously associated with fatty liver development following lipids accumulation (Oshio et al. 2021), while *KCNMA1* dysregulation has been previously reported in breast carcinoma, glioblastoma, prostate, and colorectal cancers (Basile et al. 2019).

AFB1 induction of hepatotoxicity is largely due to its capacity to generate reactive oxygen species (ROS), which can cause oxidative stress, leading to oxidative DNA damage and peroxidation of membrane phospholipids (Benkerroum 2020). Hence, effective antioxidant mechanisms are crucial in mitigating the damage caused by AFB1. To assess oxidative damage deriving from AFB1 exposure, we measured MDA, a common byproduct of lipid peroxidation, in cells treated with 1.8 µM AFB1. Surprisingly, despite the inhibition of TLR2 signaling pathway, cells lacking the *CYP1A1* gene showed significantly higher MDA levels compared to CTL ones, advising for an imbalance in the cellular antioxidant response in the CYP1A1 KO cell model, which might also explain the scant reduction of the cytotoxicity upon AFB1 exposure. This finding aligns with data from our previous study (Iori et al. 2024a), where key cytoprotective genes like *GSTA2* and *MGST1* were downregulated in BFH12 cells as a consequence of *CYP1A1* ablation. Moreover, *MGST1* mRNA was also found to be inhibited by AFB1 itself in wild-type BFH12 cells (Iori et al. 2022). Our hypothesis of a defective antioxidant response is also supported by MDA results in CYP3A74 KO cells; indeed, in these cells, the level of lipid peroxidation was comparable to CTL ones, probably thanks to MGST1, whose expression was not found to be affected by this genetic modification (Iori et al. 2024b).

Overall, despite the innovative and ground-breaking approach here used, this study showed some limitations. Because based on CRISPR/Cas9-mediated gene deletion, this study allowed solely an indirect estimation of bovine CYP1A1 and CYP3A74 contribution to AFB1 metabolism. In addition, the modifications induced by CYP KO on the cell models’ transcriptome (e.g., compensatory effects of other CYPs and the inhibition of antioxidant enzymes) might have made the interpretation of the results more difficult. Nevertheless, compared to the heterologous expression that is the most common approach employed to study the role of single enzymes in xenobiotic metabolism, the methodological approach here used provided new knowledge on AFB1 mechanistic toxicology, specifically on its biological effects in case of decreased CYP-mediated metabolism. Finally, CYP3A74 role in AFB1 8,9-epoxidation was only estimated and not confirmed by direct metabolite quantification, because of AFBO and AFB1-dihydrodiol analytical standards unavailability and AFBO high reactivity towards DNA and proteins.

### Conclusion

In conclusion, in the present study, we investigated the role of bovine CYP1A1 and CYP3A74 in AFB1 metabolism and hepatotoxicity. Molecular docking investigations suggested the involvement of CYP1A1 in AFB1 epoxidation (both 8,9-*exo-* and 8,9-*endo-*epoxide) and 9α-hydroxylation (AFM1), and the contribution of CYP3A74 in the generation of the reactive 8,9-*exo-*epoxide and the 3α-hydroxy metabolite (AFQ1). The subsequent integration of in silico results with biological in vitro data obtained after AFB1 exposure of genetically modified cells showed a major contribution of bovine CYP3A74 in AFB1 bioactivation rather than detoxification, since AFQ1 was never detected. Conversely, CYP1A1 was substantiated to be the key XME involved in AFM1 synthesis, as already observed in humans. The transcriptome analyses further corroborated the outstanding role of bovine CYP1A1 and CYP3A74 in triggering AFB1 toxicity. Indeed, CYPs deletion strongly counteracted AFB1 molecular toxic effects, since few pathways were modulated by AFB1 in engineered cells compared to BFH12-naïve ones. More in detail, a promotion of cell survival and growth, and an inhibition of TLR2 activation, postulated to trigger one of the signaling pathways linking AFB1 to oxidative stress and inflammation, were observed in CYP1A1 KO cells. On the other hand, reduced pro-inflammatory effects as well as decreased DNA damage and carcinogenesis events were observed in CYP3A74 KO cells, thus reinforcing our hypothesis of its pivotal role in AFBO formation. As a whole, the innovative and integrated approach used in the present study contributed to clarify the specific involvement of bovine CYP1A1 and CYP3A74 in the metabolism and toxicity of AFB1, and provided new knowledge on these CYPs-driven molecular events correlated with AFB1 hepatotoxicity in cattle. Moreover, these results open new perspectives in the management of bovine aflatoxicosis in Western countries affected by increasing exposure to AFB1 cause of climatic change. Indeed, the inhibition of CYP1A1 or CYP3A74 (e.g., by natural compounds as curcuminoids administered via feed) might be useful to reduce AFM1 or AFBO synthesis, limit AFM1 milk excretion and decrease AFB1 toxicity, thus preserving food safety and animal health.

### Supplementary Information

Below is the link to the electronic supplementary material.

Supplementary file1 (DOCX 4729 KB)

Supplementary file2 (DOCX 23 KB)

Supplementary file3 (XLSX 363 KB)

## Data Availability

Raw Illumina Sequencing Data have been deposited in GenBank (SRA) under the BioProject ID PRJNA1068490.
