# Supplementary material for: New insights into aflatoxin B1 mechanistic toxicology in cattle liver: an integrated approach using molecular docking and biological evaluation in CYP1A1 and CYP3A74 knockout BFH12 cell lines
Source: Arch Toxicol. 2024 Jun 4;98(9):3097–108. doi: 10.1007/s00204-024-03799-y (PMC11324698; doi:10.1007/s00204-024-03799-y)
Supplement: Supplementary file 1 — Supplementary file1 (DOCX 4729 KB) [file 204_2024_3799_MOESM1_ESM.docx]

**New insights into Aflatoxin B1 mechanistic toxicology in cattle liver: an integrated approach using molecular docking and biological evaluation in CYP1A1 and CYP3A74 knockout BFH12 cell lines**

Silvia Iori^a^, Maija Lahtela-Kakkonen^b^, Caterina D’ Onofrio^a^, Federica Maietti^a^, Greta Mucignat^a^, Anisa Bardhi^c^ Andrea Barbarossa^c^, Anna Zaghini^c^, Marianna Pauletto^a^, Mauro Dacasto^a^ & Mery Giantin^a,*^

^a^Department of Comparative Biomedicine and Food Science, University of Padua, Viale dell’Università 16, Legnaro, 35020 Padua, Italy

^b^University of Eastern Finland, School of Pharmacy, Yliopistonrinne 3, 70210 Kuopio, Finland

^c^Department of Veterinary Medical Sciences, Alma Mater Studiorum University of Bologna, Via Tolara di Sopra 50, Ozzano dell’Emilia, 40064 Bologna, Italy

*corresponding author at: Department of Comparative Biomedicine and Food Science, University of Padua, Viale dell’Università 16, Legnaro, 35020 Padua, Italy.
E-mail address: mery.giantin@unipd.it (M. Giantin).

**Supplementary Figures**

**a**

**
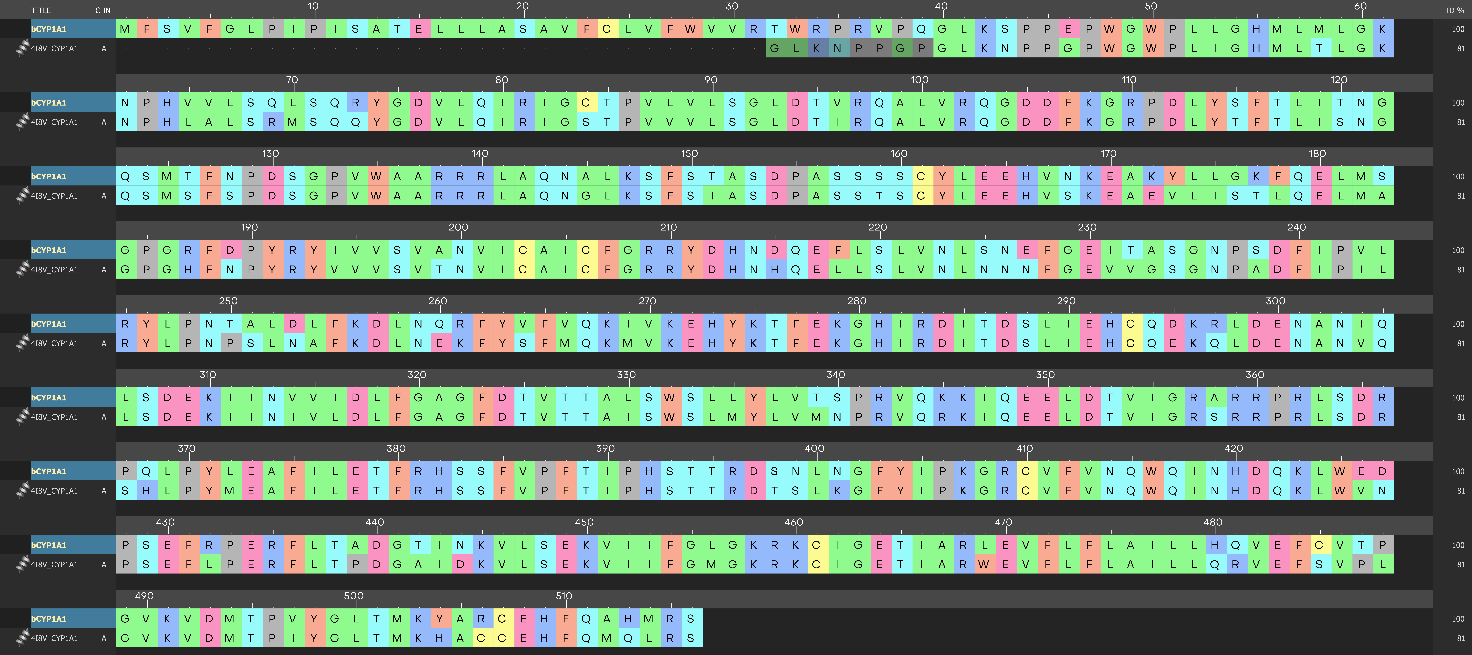
**

**b**

**
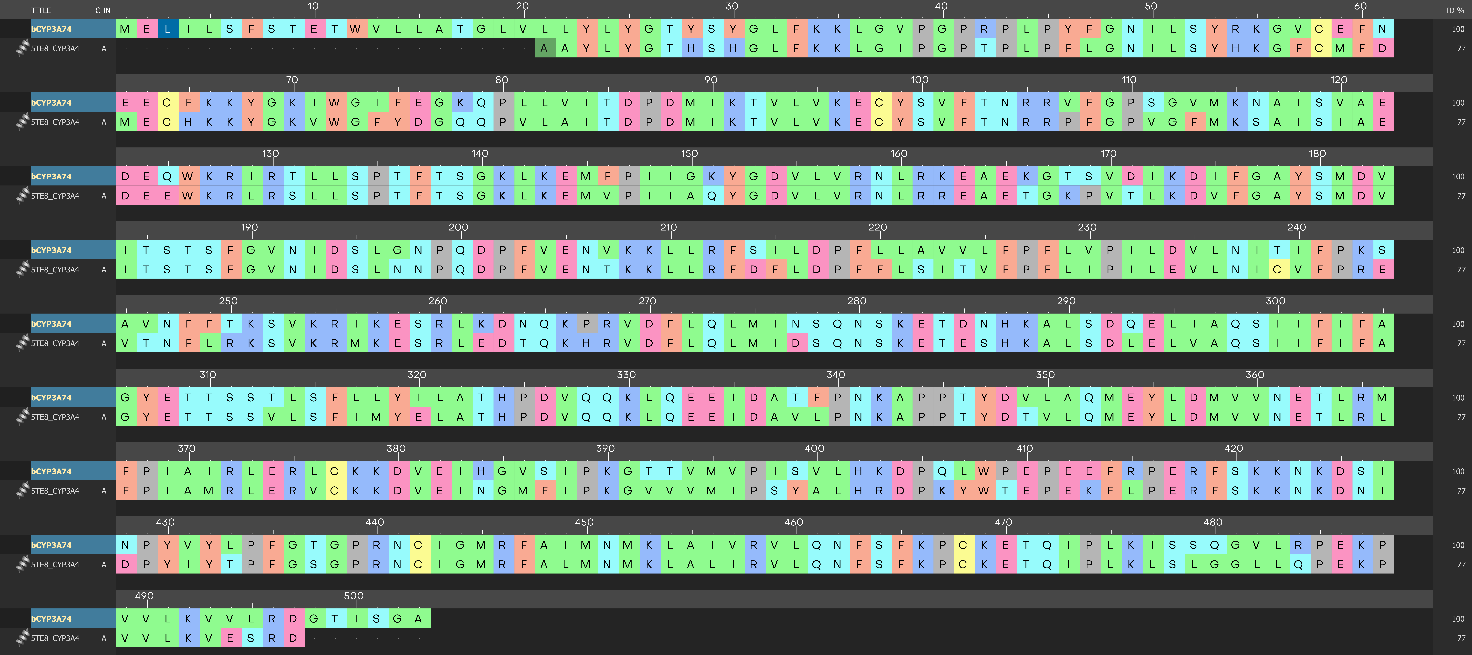
**

**Supplementary Fig. 1**. Sequence alignment between bovine CYP1A1 (bCYP1A1) and human CYP1A1 (**a**) and between bovine CYP3A74 (bCYP3A74) and human CYP3A4 (**b**).

**a**

**
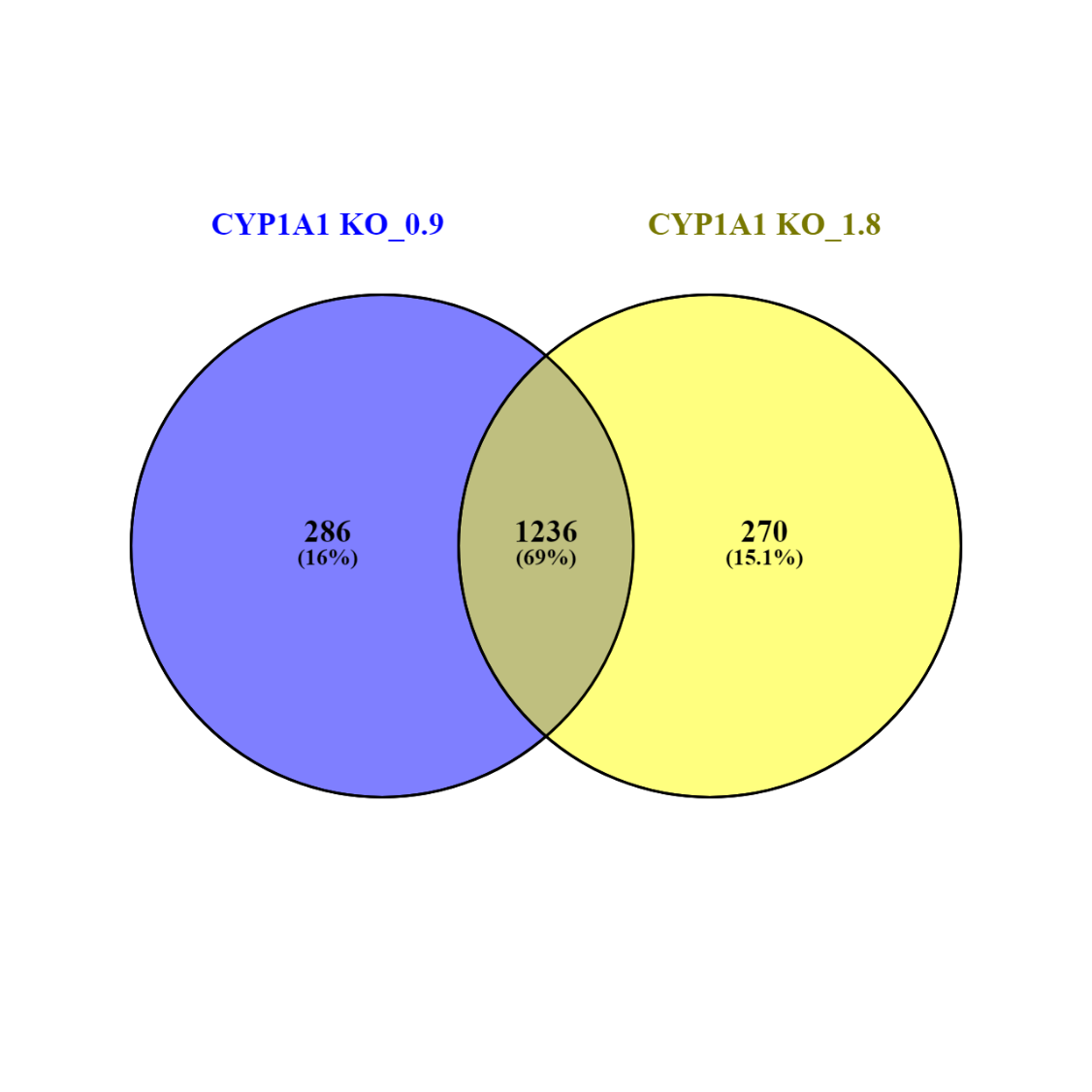
**

**b**

**
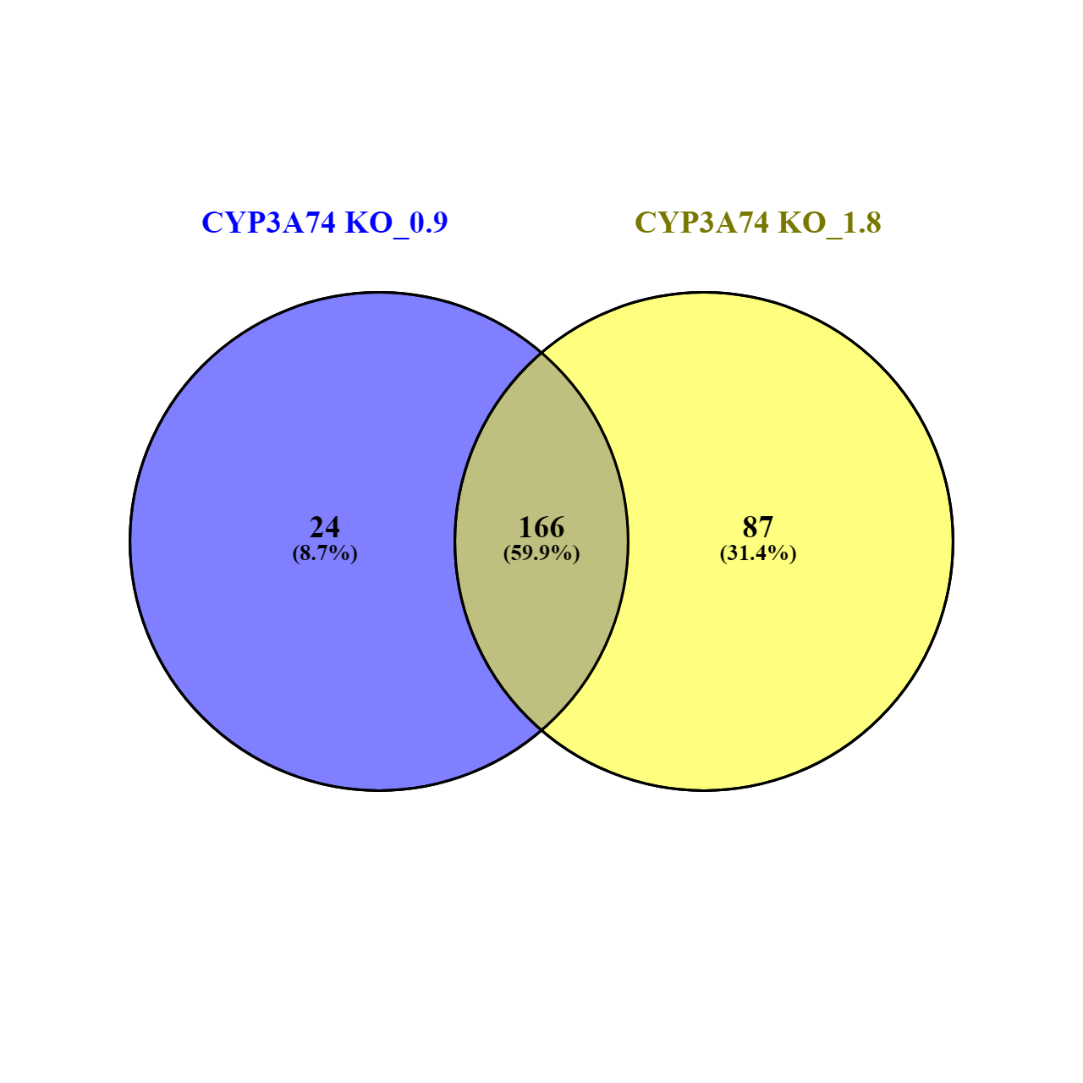
**

**Supplementary Fig. 2**. Venn diagram reporting number and percentages of unique and in common DEGs among the different AFB1 treatment conditions (i.e., 0.9 µM and 1.8 µM) in CYP1A1 vs CTL (**a**) and CYP3A74 KO vs CTL (**b**) cells.

**a**


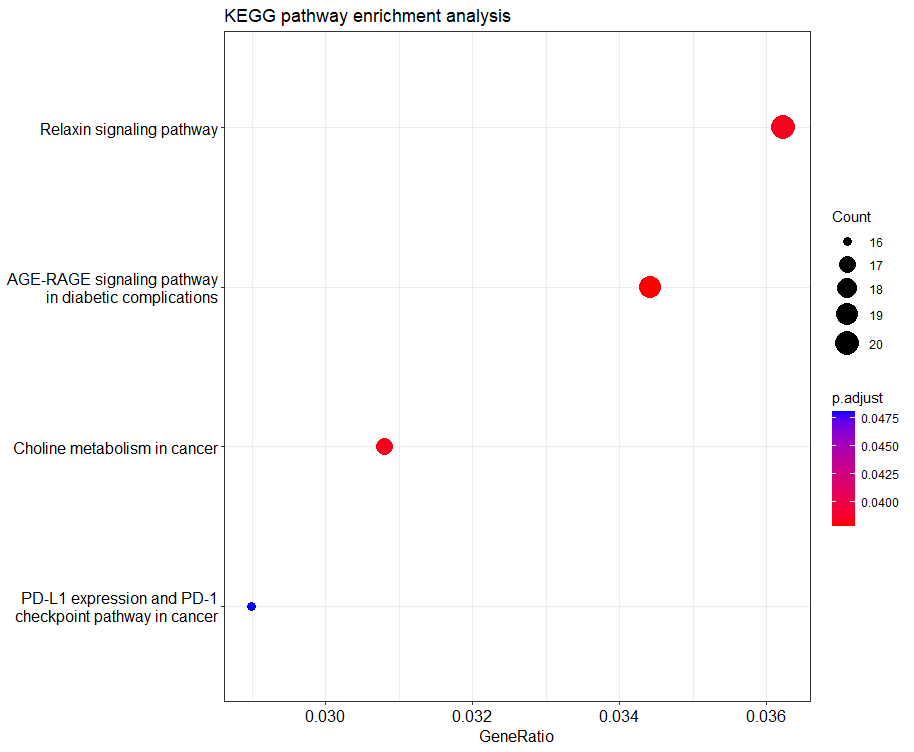


**b**

**
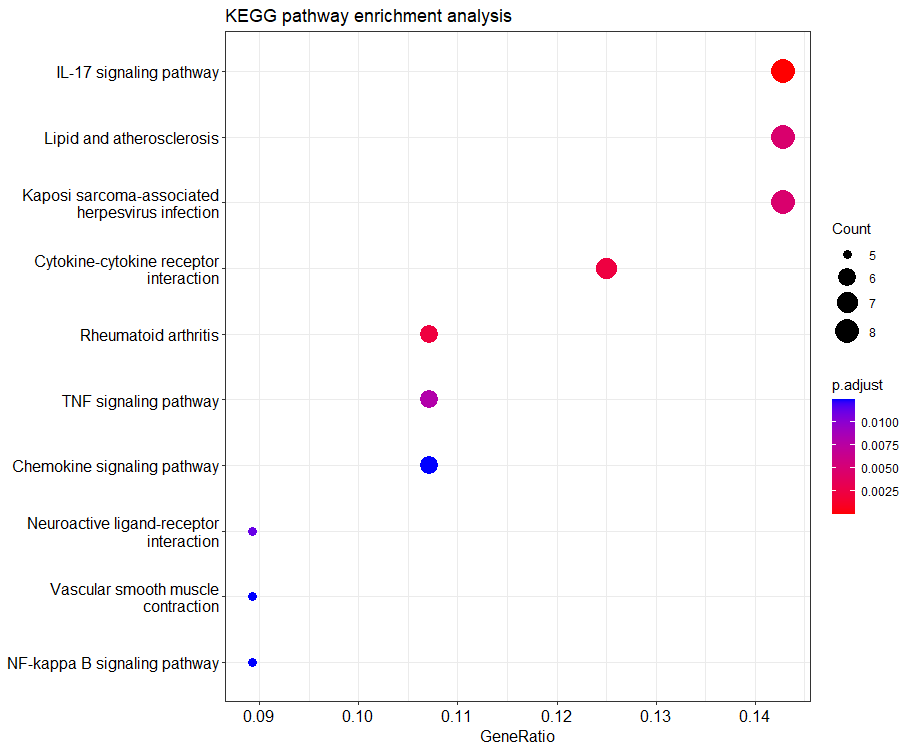
**

**Supplementary Fig. 3** KEGG enrichment analysis conducted on DEGs between CYP1A1 KO (**a**) and CYP3A74 (**b**) and CTL cells upon AFB1 exposure. Count (dot size) represents the number of DEGs enriched in a certain pathway. The colour gradient represents the adjusted significance level (p. adjusts), according to the Benjamin-Hochberg method.


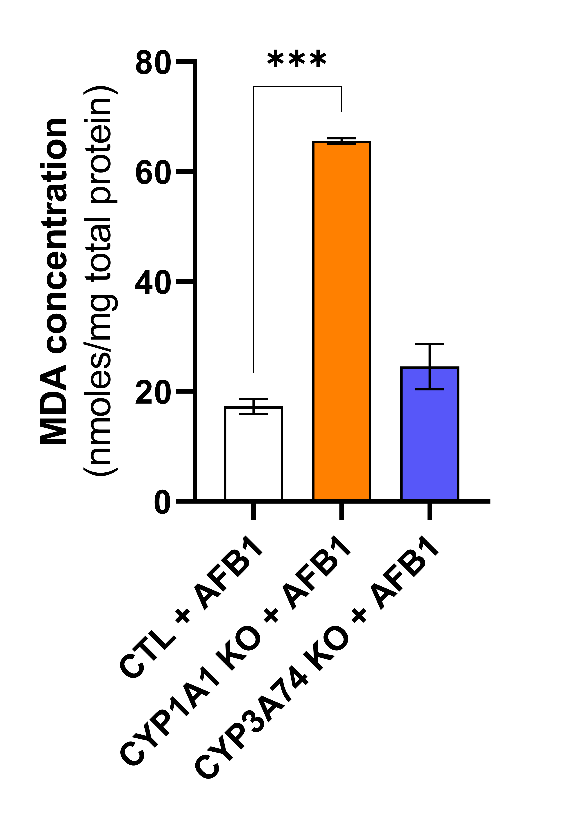


**Supplementary Fig. 4.** Amount of malondialdehyde (MDA) in CTL, CYP1A1 KO and CYP3A74 KO cells exposed to 1.8 µM AFB1. Data are expressed as nmoles of MDA per mg of total protein, as the mean ± SEM of three biological replicates. Statistical analysis: one-way ANOVA followed by Dunnett’s multiple comparisons test; ***: *p* < 0.001.


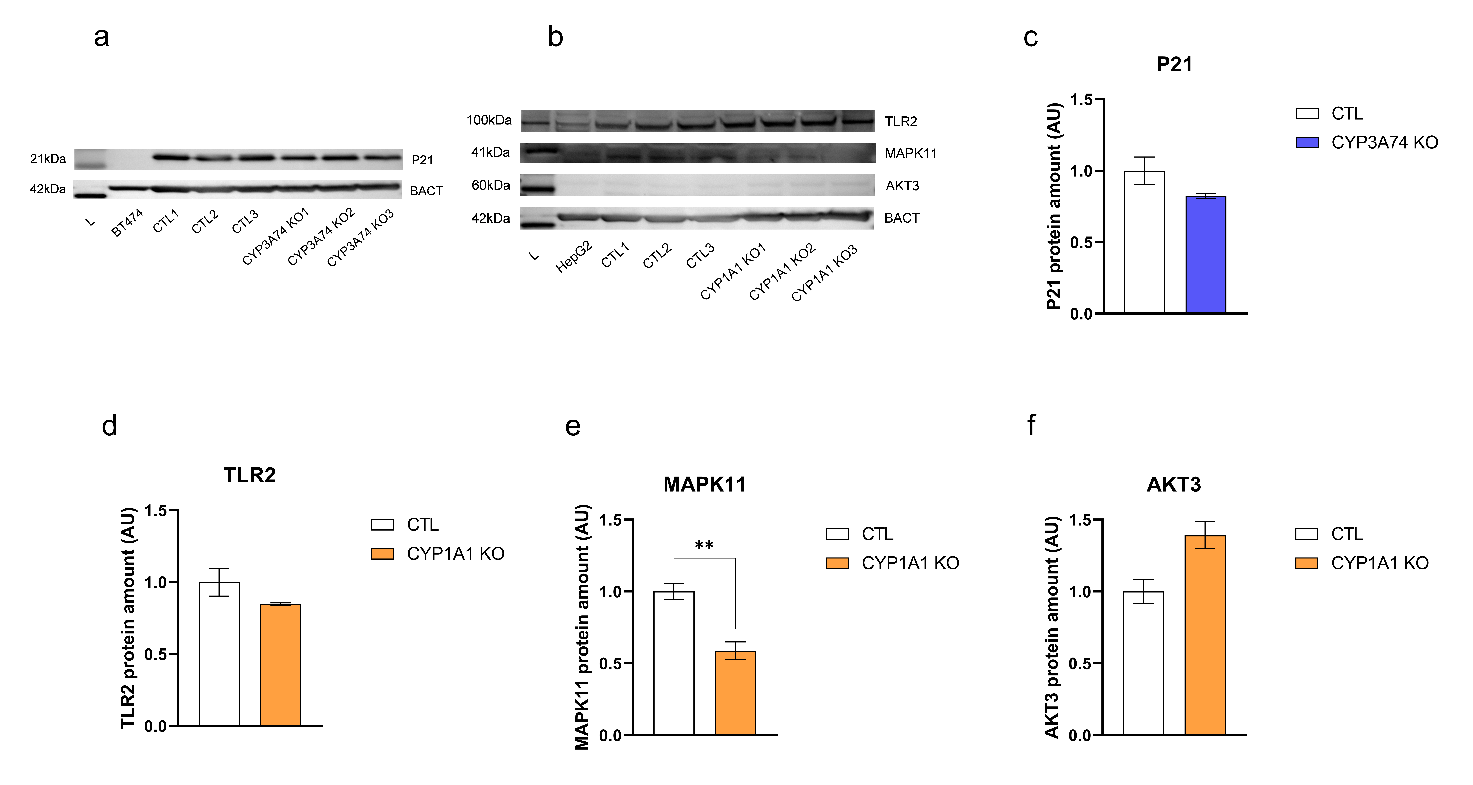


**Supplementary Fig. 5.** Immunoblotting of P21 (**a**), TLR2, MAPK11 and AKT3 (**b**) in CTL and KO cells treated with 1.8 uM AFB1; BACT was used as loading control. Densitometric analysis of P21 (**c**), TLR2 (**d**), MAPK11 (**e**) and AKT3 (**f**) immunoblottings; data are expressed in AU as the mean ± SEM of three biological replicates. Statistical analysis: unpaired t-test with Welch’s correction. **: *p*<0.01.
