# Supplementary material for: New insights into aflatoxin B1 mechanistic toxicology in cattle liver: an integrated approach using molecular docking and biological evaluation in CYP1A1 and CYP3A74 knockout BFH12 cell lines
Source: Arch Toxicol. 2024 Jun 4;98(9):3097–108. doi: 10.1007/s00204-024-03799-y (PMC11324698; doi:10.1007/s00204-024-03799-y)
Supplement: Supplementary file 2 — Supplementary file2 (DOCX 23 KB) [file 204_2024_3799_MOESM2_ESM.docx]

**New insights into Aflatoxin B1 mechanistic toxicology in cattle liver: an integrated approach using molecular docking and biological evaluation in CYP1A1 and CYP3A74 knockout BFH12 cell lines**

Silvia Iori^a^, Maija Lahtela-Kakkonen^b^, Caterina D’ Onofrio^a^, Federica Maietti^a^, Greta Mucignat^a^, Anisa Bardhi^c^, Andrea Barbarossa^c^, Anna Zaghini^c^, Marianna Pauletto^a^, Mauro Dacasto^a^ & Mery Giantin^a^,*

^a^Department of Comparative Biomedicine and Food Science, University of Padua, Viale dell’Università 16, Legnaro, 35020 Padua, Italy

^b^University of Eastern Finland, School of Pharmacy, Yliopistonrinne 3, 70210 Kuopio, Finland

^c^Department of Veterinary Medical Sciences, Alma Mater Studiorum University of Bologna, Via Tolara di Sopra 50, Ozzano dell’Emilia, 40064 Bologna, Italy

*corresponding author at: Department of Comparative Biomedicine and Food Science, University of Padua, Viale dell’Università 16, Legnaro, 35020 Padua, Italy.
E-mail address: mery.giantin@unipd.it (M. Giantin).

Supplementary file

Materials and Methods page 2

References page 4

Materials and Methods

Homology modelling and molecular docking of AFB1 into CYP1A1 and CYP3A74 models

Bovine CYP1A1 model was obtained starting from the x-ray human CYP1A1 in complex with alpha-naphthoflavone (PDB:4I8V, R=2.6 Å) (Walsh et al. 2013), while the homology model of bovine CYP3A74 was built using as template the crystal structure of midazolam bound to human CYP3A4 (PDB:5TE8, R=2.7 Å) (Sevrioukova and Poulos 2017). Molecular modelling was performed using the Schrödinger Maestro version 12.8 (Small-Molecule Drug Discovery Suite 2021-2, Schrödinger, LLC, New York, NY). Templates were firstly prepared with Protein Preparation Wizard. Bond orders and hydrogens were added, and missing chains as well as missing loops were filled using Prime module. Chain A was applied in the study and het states were generated using Epik (pH 7±2). The heme iron was set as Fe^3+^ and it was connected via zero-order bonds to the conserved cysteine sulphur and to the four heme nitrogens. The hydrogen bonds were assigned using PROPKA (pH 7.0) and waters having less than 3 H-bonds to non-waters were removed (default parameters). Then, protein minimization was conducted using the OPLS2005 force field, with heavy atom converging 0.30 Å RMSD. For homology modelling, sequence alignment was performed using a Smith-Waterman algorithm in Multiple Sequence Viewer/Editor; then, the Build Homology Model was used to obtain the 3D model of CYP1A1 and CYP3A74. For each obtained model, Structurally Conserved Regions (SCRs) were checked and Ramachandran plot was built to evaluate the quality of homology models. By means of LigPrep, co-crystalized ligands and AFB1 (PubChem CID: 186907) were prepared and minimized using OPLS4 force filed. Compounds were ionized at pH 7 +/- 2 and desalts and tautomers were generated using Epik. The binding site of each CYP was set with Receptor Grid Generation tool based on the binding of co-crystallized ligand, i.e. alpha-naphthoflavone and midazolam for CYP1A1, and for CYP3A4, respectively. Co-crystallized ligands of templates were docked in the respective deriving models to assure a correct binding mode for the target (i.e., AFB1). Then, AFB1 was docked into the models. Specifically, co-crystallized ligands as well as AFB1 were docked into the CYPs using Glide SP (Scaling factor of van der Waals radii was set 0.60).

LC-MS/MS quantification of AFB1, AFM1, AFL and AFQ1

AFB1 and its metabolites (AFM1, AFL, and AFQ1) were quantified by LC-MS/MS in the medium of CTL and KO cells incubated with 0.9 and 1.8 µM AFB1. Samples were thawed at room temperature (20°C) and mixed by vortexing for 30 s. Then, 20 µL of sample were transferred into a LC vial containing 180 µL of ultrapure water with 0.1% formic acid, and 20 µL of the internal standard AFM2 (200 ng/mL in acetonitrile). Finally, 5 µL were injected in the analytical system. The LC consisted of a Waters Acquity UPLC binary pump, equipped with an Acquity BEH C18 (50 × 2.1 mm, 1.7 µm) reversed-phase column, kept at 40°C (Waters, Milford, MA, USA). Chromatographic separation was obtained in a 4 min run under programmed conditions, with a variable mixture of 0.1% formic acid in water and acetonitrile flowing at 0.3 mL/min. The detector was a Waters Xevo TQ-S Micro triple quadrupole mass spectrometer (Waters, Milford, MA, USA), operating in positive electrospray ionization (ESI+) with 3.5 kV capillary voltage. Source temperature was 150°C and desolvation temperature was 600°C; desolvation and cone gas flow were 900 and 50 L/h, respectively. For each analyte, the following specific transitions (with relative Cone Voltage and Collision Energy values) were monitored: 313.18 > 241.18 *m/z* (CV 52 V; CE 33 eV) and 313.18 > 285.03 *m/z* (CV 52 V; CE 18 eV) for AFB1; 297.20 > 225.24 *m/z* (CV 52V; CE 31 eV) and 297.20 > 268.78 *m/z* (CV 52 V; CE 15 eV) for AFL; 329.17 > 229.19 *m/z* (CV 52 V; CE 35 eV) and 329.17 > 273.18 *m/z* (CV 52 V; CE 18 eV) for AFM1; 329.17 > 206.12 *m/z* (CV 52 V; CE 18 eV) and 329.17 > 177.29 *m/z* (CV 52 V; CE 28 eV) for AFQ1; 331.19 > 273.18 (CV 52; CE 16 V) for AFM2. Data acquisition and analysis were carried out with MassLynx 4.2 software (Waters, Milford, MA, USA). The linearity of the method was assessed (r^2^ > 0.99) over the 0.045-1.8 µM range for AFB1 and the 0.001-0.06 µM range for the metabolites through matrix-matched calibrators prepared and analysed in parallel to each batch of samples. The lower limit of quantification (LLOQ), intended as the lowest measured concentration providing a signal/noise ratio above 10:1, was 0.001 µM for all analytes. The recovery was within 90-105 % for all compounds.

RNA-sequencing data analyses

Raw reads were trimmed using the BBDuk program (BBTools suite) and then mapped against the *Bos taurus* ARS-UCD1.2 reference genome (Iori et al., 2024a). The differential expression analysis was conducted using the EdgeR Bioconductor package (Robinson et al. 2010). Pair-wise comparisons were carried out to highlight transcriptional changes induced by AFB1 exposure in KO and CTL cells, setting a False Discovery Rate (FDR) of 5%. ClusterProfiler package (Yu et al. 2012) was then implemented in R environment to functionally interpret significant differentially expressed genes (DEGs) through the Kyoto Encyclopedia of Genes and Genomes (KEGG) over-representation test.

Immunoblotting

Fifteen µg of total proteins were separated on NuPAGE® Novex® 4–12% Bis–Tris Gels by using the XCell SureLock™ Mini-Cell electrophoresis system (Invitrogen, Eugene, OR, USA), and then transferred onto nitrocellulose filters as previously described (Zancanella et al., 2012). Proteins from HepG2 and BT474 human cell lines were used as positive controls. Membranes were incubated with anti-ACTB (1:6000 final concentration, 2 hrs), TLR2 (1:1000, overnight), MAPK11 (1:1000, overnight), AKT3 (1:3000, overnight) and P21 (1:500, overnight) primary antibodies, then with horseradish peroxidase-conjugated goat anti-rabbit (1:6000, 1.5 hrs) for ACTB, TLR2, AKT3 and P21 or anti-mouse IgG (1:5000, 1.5 hrs) for MAPK11. The specific proteins were detected using SuperSignal® West Pico chemiluminescence substrate (Pierce, Life Technologies, Foster City, CA, USA) according to the manufacturer’s instructions. Immunopositive bands were captured by the iBright Imaging Systems (iBright FL1500, Thermo Fisher Scientific, Waltham, MA, USA) and their Integrated Optical Density (IOD) was acquired by means of ImageJ 1.44p image analysis software. For the semi-quantification analysis, IOD of each sample was normalized to the IOD of the loading control (ACTB).

**References**

Iori S, D’Onofrio C, Laham-Karam N, et al (2024a) Establishment and characterization of cytochrome P450 1A1 CRISPR/Cas9 Knockout Bovine Foetal Hepatocyte Cell Line (BFH12). Cell Biol Toxicol 40:18. https://doi.org/10.1007/s10565-024-09856-7

Robinson MD, McCarthy DJ, Smyth GK (2010) edgeR: a Bioconductor package for differential expression analysis of digital gene expression data. Bioinformatics 26:139–140. https://doi.org/10.1093/bioinformatics/btp616

Sevrioukova IF, Poulos TL (2017) Structural basis for regiospecific midazolam oxidation by human cytochrome P450 3A4. P Proc Natl Acad Sci U S A 114:486–491. https://doi.org/10.1073/pnas.1616198114

Walsh AA, Szklarz GD, Scott EE (2013) Human cytochrome P450 1A1 structure and utility in understanding drug and xenobiotic metabolism. J Biol Chem 288:12932–12943. https://doi.org/10.1074/JBC.M113.452953

Yu G, Wang L-G, Han Y, He Q-Y (2012) ClusterProfiler: an R Package for Comparing Biological Themes Among Gene Clusters. OMICS 16:284–287. https://doi.org/10.1089/omi.2011.0118

Zancanella V, Giantin M, Lopparelli RM, et al (2012) Constitutive Expression and Phenobarbital Modulation of Drug Metabolizing Enzymes and Related Nuclear Receptors in Cattle Liver and Extra-Hepatic Tissues. Xenobiotica 42:1096–1109. https://doi.org/10.3109/00498254.2012.694493
